# Comparing Post-operative Anticoagulation: Rivaroxaban and Apixaban Demonstrate Similar Clot Strengths

**DOI:** 10.1016/j.ejvs.2025.01.047

**Published:** 2025-02-05

**Authors:** Aaliya Ashik, Adriana Rodriguez, Lois Owolabi, Isabella Cieri, Shiv Patel, Anahita Dua

**Affiliations:** a The University of Manchester, School of Medical Sciences, Manchester, UK; b Division of Vascular and Endovascular Surgery, Massachusetts General Hospital, Boston, MA, USA; c Harvard Medical School, Cambridge, MA, USA

**Keywords:** Atherothrombosis, Endovascular, Factor Xa inhibitors, Open vascular surgery, Peripheral arterial disease, Post-operative care

Patients with peripheral arterial disease (PAD) are susceptible to major adverse limb events, such as amputation or chronic limb threatening ischaemia, following revascularisation procedures.^1^ Current European Society for Vascular Surgery (recommendations 78 and 80) and American College of Cardiology and American Heart Association multisociety guidelines recommend dual pathway inhibition for post-operative thromboprophylaxis^2^ based on results from the VOYAGER (Vascular outcomes study of acetylsalicylic acid along with rivaroxaban in endovascular or surgical limb revascularisation) PAD trial to reduce the risk of major adverse limb events.^1,3^ Limited data have compared Factor Xa inhibitors in patients with PAD, most likely because of the increased standardisation of clinical prescribing.

This study compared multiple anticoagulant regimens because of the heterogeneity of post-operative thromboprophylaxis prescribing in the USA. Apixaban was felt to be an important anticoagulant to study, given that many patients with PAD take apixaban for other longstanding medical issues;^4^ therefore, it may be included in their post-operative thromboprophylaxis. This study used thrombo-elastography with platelet mapping (TEG-PM) to compare the effect of rivaroxaban and apixaban on clot strength. Unlike traditional coagulation tests, such as prothrombin time and partial thromboplastin time, TEG-PM uses dual pathway metrics to provide coagulation profiles based on real time measurements of viscoelastic clot properties. The assay of interest reveals the maximum clot strength exclusively resulting from adenosine diphosphate activated platelets (MA_ADP_).^5^ A study by Suarez Ferreira *et al.*^6^ determined that MA_ADP_ values > 42 mm were associated with a higher risk of thrombosis in patients with PAD and could have predictive value.

Patients scheduled for lower extremity revascularisation procedures (endovascular and open) between 8 April 2021 and 18 December 2023 at a large tertiary institution were enrolled prospectively and followed clinically. The TEG-PM assays taken one month after surgery were analysed. Inclusion criteria were patients who had a successful revascularisation procedure, were taking apixaban or rivaroxaban, and had antiplatelet regimens consisting of aspirin and or clopidogrel. Successful revascularisation was defined as restoring adequate blood flow to the ischaemic limb. The study protocol and consent form were approved by the Institutional Review Board (number 2022P001918). Blood samples were first stratified based on the anticoagulant type and subsequently on the antiplatelet regimen (aspirin, clopidogrel, or dual antiplatelet therapy). Some patients were prescribed triple therapy if they had a higher risk of thrombosis or were undergoing redo procedures.

Sixty eight TEG-PM samples were analysed. Twenty two patients (32%) took rivaroxaban and 46 (68%) took apixaban. The results are displayed as median and interquartile range (IQR). Mann–Whitney *U* testing was used to compare anticoagulant groups. Fisher’s exact test and chi squared tests were used to compare categorical variables. No statistically significant difference was found between the rivaroxaban and apixaban groups in demographics (age 69 years [IQR], 95% confidence interval [CI] 61.7 – 72.7 *vs.* 71 years [IQR], 95% CI 63.6 – 71.6, *p* = .77; male 68% *vs.* 78%; *p* = .37; white 86% *vs.* 80%, *p* = .55). No statistically significant difference was observed in the comorbidities (diabetes mellitus 59% *vs.* 65%, *p* = .62; hypertension 82% *vs.* 89%, *p* = .46; hyperlipidaemia 86% *vs.* 83%, *p* > .99; normal renal status 50% *vs.* 33%, *p* = .17). No statistically significant difference was found in MA_ADP_ values despite different anticoagulant dosages within the rivaroxaban (2.5 mg *vs.* 20 mg, *p* = .68; 20 mg *vs.* 10 mg, *p* = .74; 2.5 mg *vs.* 10 mg, *p* = .95) and apixaban groups (2.5 mg *vs.* 5 mg, *p* = .59).

No statistically significant difference in the MA_ADP_ was found between rivaroxaban and apixaban before substratifying for antiplatelet use. Similarly, substratification by antiplatelet regimen also showed no statistically significant difference in MA_ADP_ between rivaroxaban and apixaban (Table 1). In rivaroxaban users, no statistically significant difference was found in the MA_ADP_ values between single and dual antiplatelet therapy users (52.1 mm, 95% CI 30.2 – 61.7 *vs.* 45.0 mm, 95% CI 27.7 – 47.9, *p* = .29). Similarly, no statistically significant difference was found between antiplatelet regimen cohorts within the apixaban group (42.9 mm, 95% CI 37.3 – 49.5 *vs.* 47.2 mm, 95% CI 31.3 – 54.3, *p* = .98). When comparing the incidence of thrombotic events between rivaroxaban and apixaban groups, no statistically significant differences were found (18% *vs.* 22%, *p* = .99). No statistically significant difference was found in MA_ADP_ values between thrombotic event groups (non-event 35.6 mm, 95% CI 8.2 – 63.1 *vs.* event 52.4 mm, 95% CI 33.6 – 61.3, *p* = .24).

Interestingly, this study showed no statistically significant difference in MA_ADP_ values between patients taking rivaroxaban and apixaban. This finding was seen regardless of the antiplatelet regimen. Given the low incidence of thrombotic (*n* = 14) and bleeding events (*n* = 2), definitive conclusions regarding anticoagulation regimens could not be drawn. No statistically significant difference was seen in the MA_ADP_ values between single and dual antiplatelet therapy subgroups in patients taking apixaban or rivaroxaban.

Limitations of this study included: the small sample size and subgroup comparisons restricted the power and generalisability of results, and a lack of a matched control group impeded the ability to evaluate the true effect of the anticoagulation regimen.

No statistically significant difference was seen in MA_ADP_ between patients taking rivaroxaban compared with apixaban. Based on previous results from Suarez Ferreira *et al.*,^6^ this may suggest that both medications are equally effective for thromboprophylaxis in the PAD population. These results suggest that there may be no indication for switching between medications to abide by guidelines. This study was hypothesis generating and deserves further research to draw definitive conclusions.

## Figures and Tables

**Table 1.** Comparison of adenosine diphosphate activated platelets (MA_ADP_) values in patients with peripheral arterial disease taking rivaroxaban and apixaban by antiplatelet regimen.

Antiplatelet regimen	Rivaroxaban group			Apixaban group			*p* value*
	Median	*n*	95% CI	Median	*n*	95% CI	
Dual antiplatelet therapy or single antiplatelet therapy (*n* = 68)	49.6	22	33.0–49.3	45.1	46	38.0–48.4	.74
Dual antiplatelet therapy (*n* = 26)	45.0	13	27.7–47.9	47.2	13	31.3–54.3	.55
Aspirin (*n* = 25)	50.7	6	24.5–68.2	42.9	19	37.5–51.1	.78
Clopidogrel (*n* = 17)	58.3	3	−15.5–105.8	45.9	14	29.8–54.5	

CI = confidence interval

* By Mann–Whitney *U* test.
